# Supplementary figures and images for: Collagen pre-strain discontinuity at the bone—Cartilage interface
Source: PLoS One. 2022 Sep 15;17(9):e0273832. doi: 10.1371/journal.pone.0273832 (PMC9477506; doi:10.1371/journal.pone.0273832)

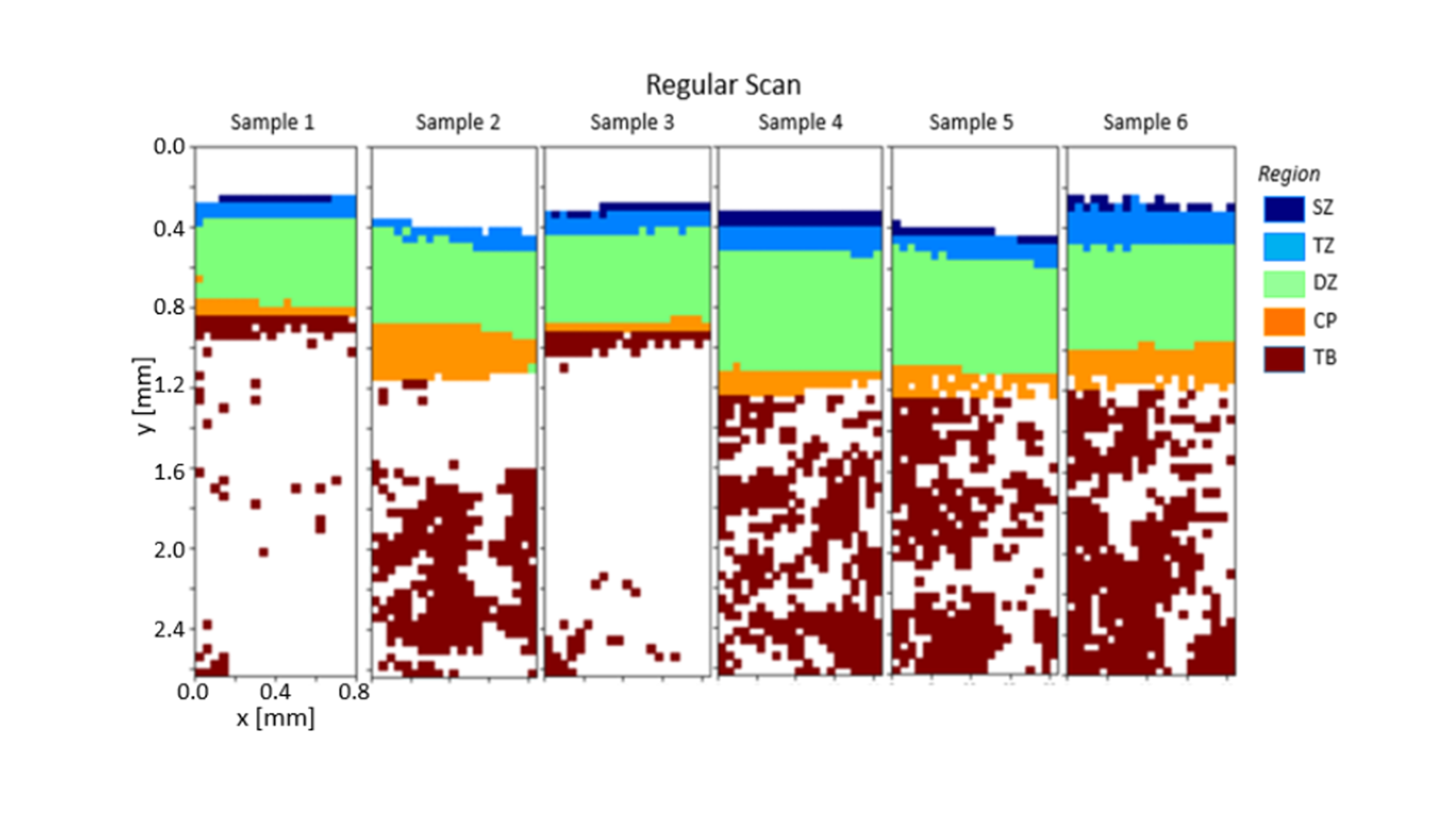

Supplement: S1 Fig — Representation of the region classification into SZ, TZ, DZ, CP and TB, for the 6 samples used in the analysis. Note that Sample 2 does not have an observable superficial (SZ) zone. The colour indicates the type of tissue region across BCU. (TIF) [file pone.0273832.s001.tif]

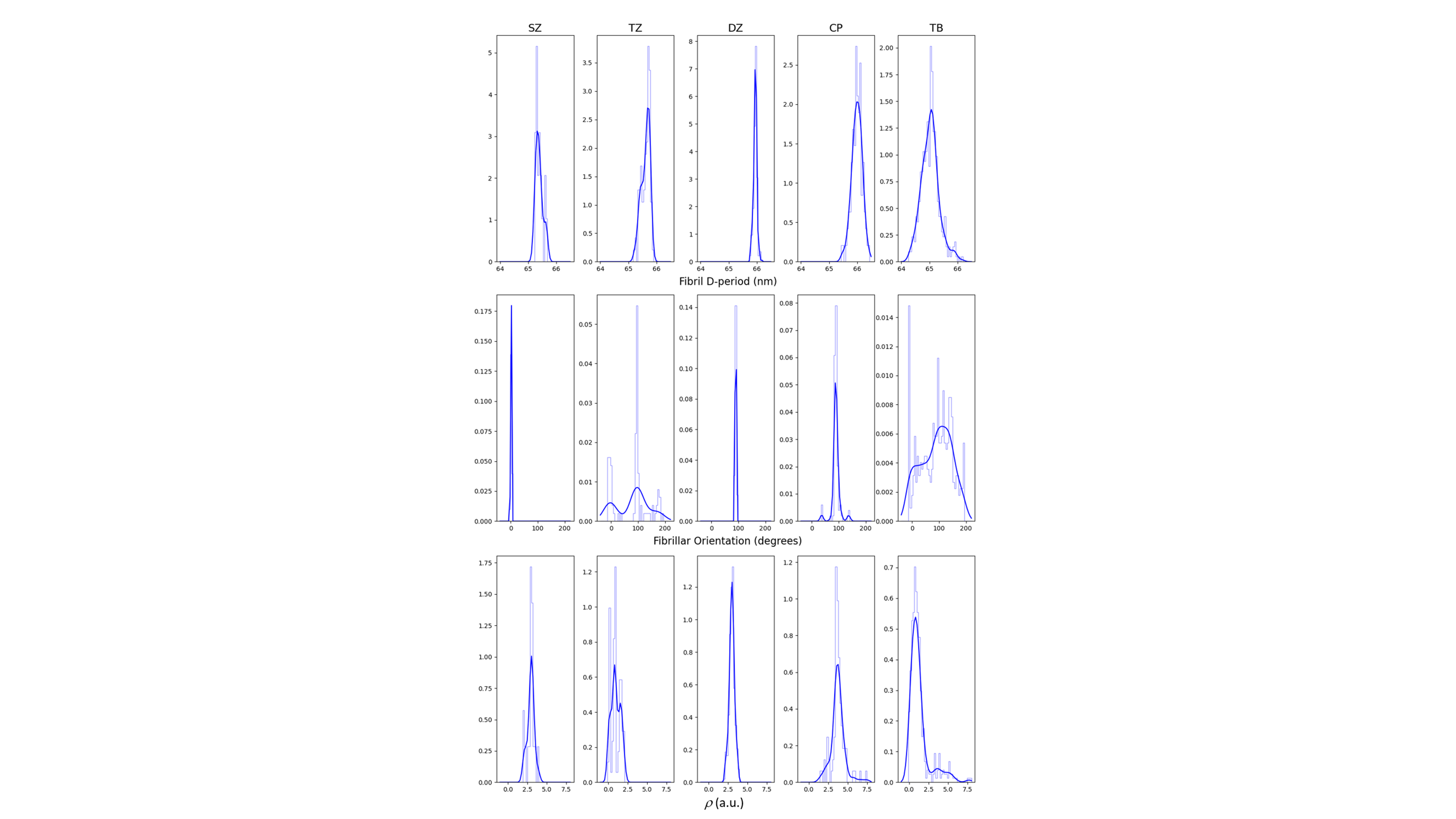

Supplement: S2 Fig — Top to bottom: Histograms of the D-period, fibril orientation and ρ for the representative sample in Figs 2 and 3, for (left to right) the SZ, TZ, DZ, CP and TB tissue regions, respectively. The smooth lines are the associated kernel density distribution estimates and are shown for visualisation only. (TIF) [file pone.0273832.s002.tif]

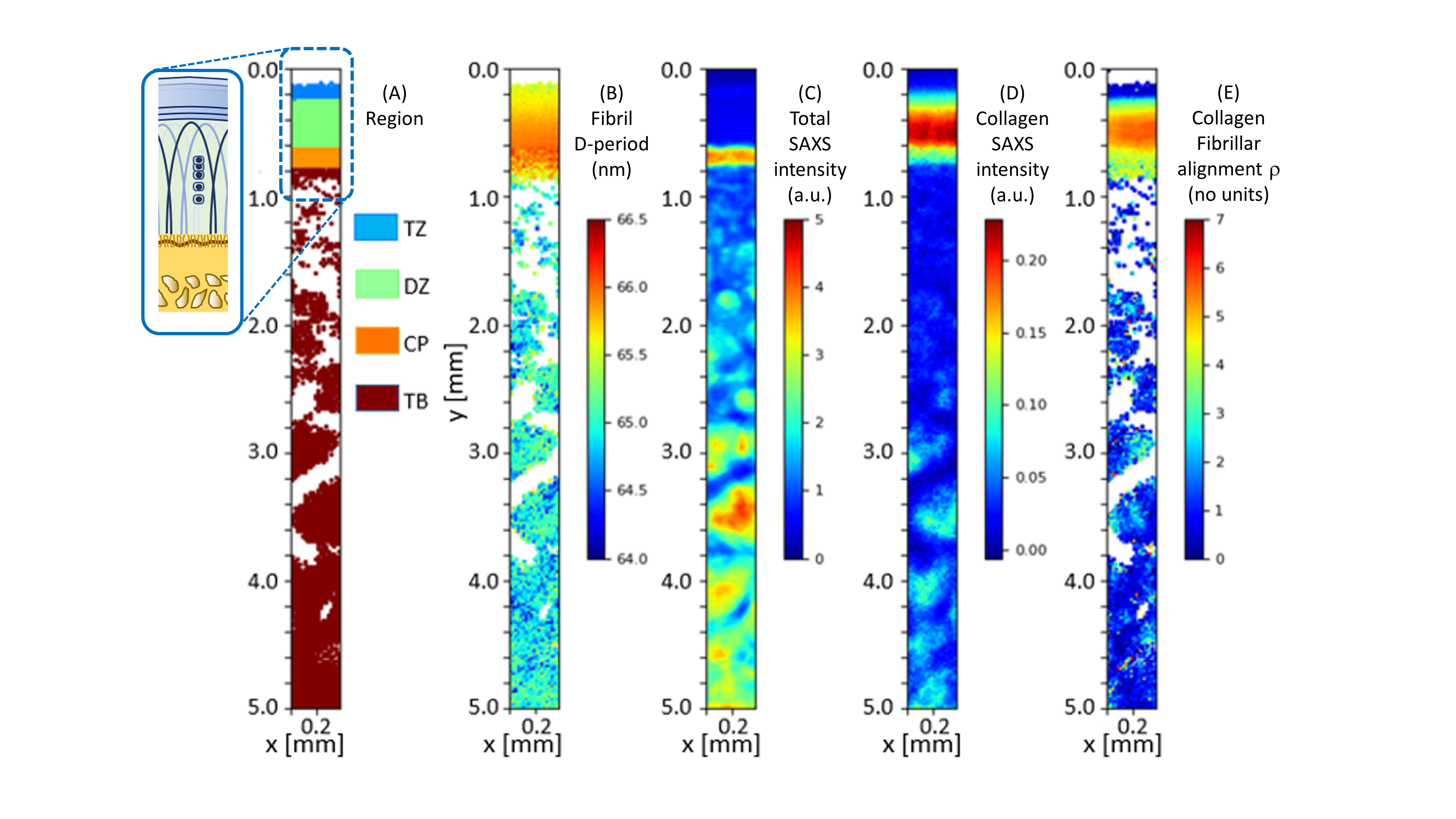

Supplement: S3 Fig — Colour map of the depth-wise variation in SAXS derived parameters across bovine bone-cartilage core of 5mm length and 2mm diameter for a full-length scan. This single sample was scanned across a greater depth in the trabecular bone but is otherwise similar to the samples imaged in Figs 2 and 3 in the main text. Step size of 20 microns, sample size ~0.38mm width, 5mm length. In this sample we were unable to resolve the thin superficial zone (SZ) at the top. Colour plots display: (A) Regions TZ: transitional zone, DZ: deep zone, CP: calcified plate, and TB: trabecular bone (as in Fig 1A, main text), (B) D-period (nm), reflecting collagen pre-strain, (C) total SAXS intensity (a.u.); areas of high intensity correspond to mineral-dense regions, (D) Total SAXS intensity from the background-corrected meridional collagen peak intensity; here, high intensity is observed in articular cartilage and (E) degree of orientation ρ (a.u.), showing high values in the deep zone (DZ), intermediate values in the calcified plate (CP) and transitional zone (TZ), and low values in trabecular bone (TB). (TIF) [file pone.0273832.s003.tif]

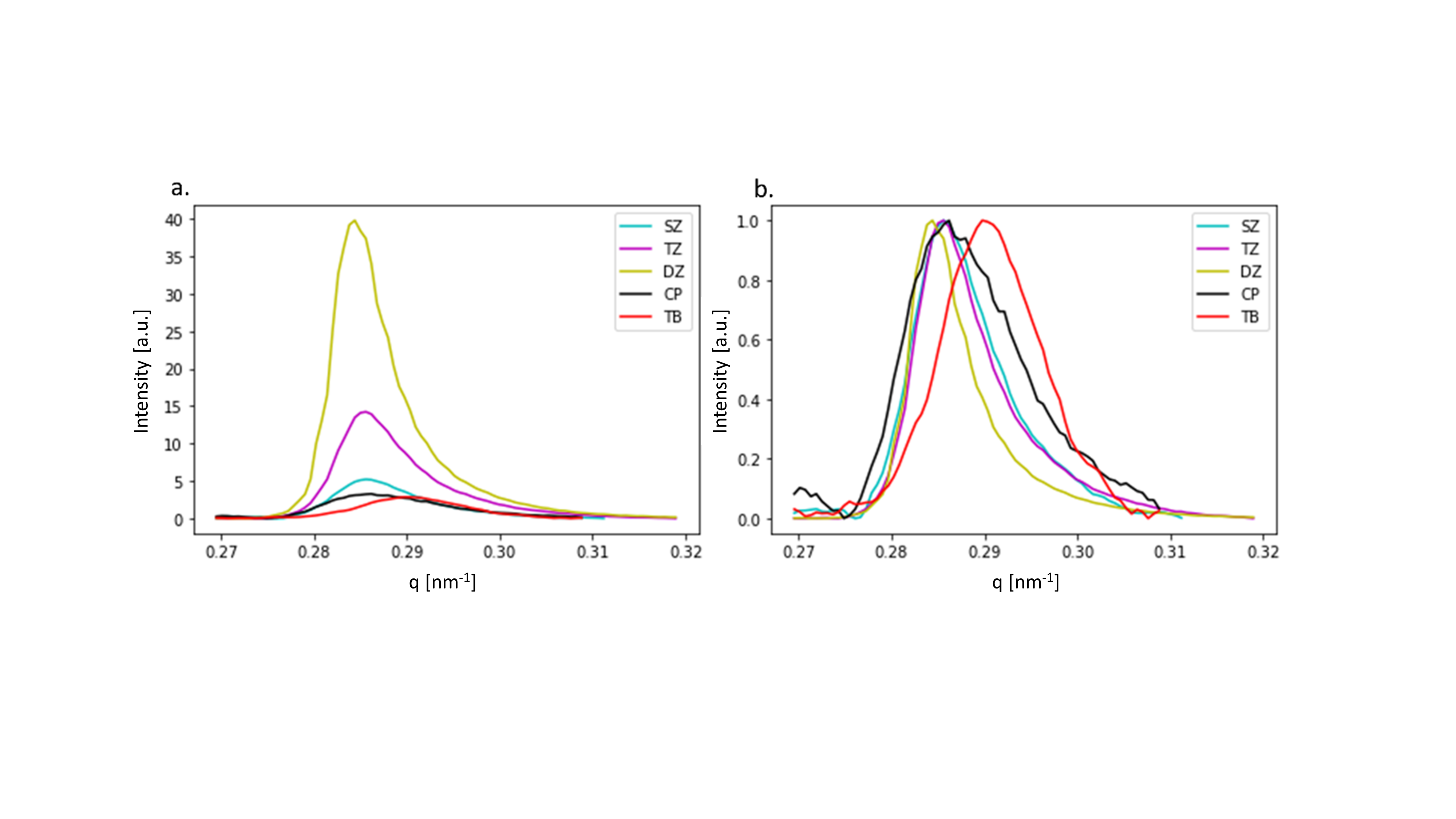

Supplement: S4 Fig — (A) Azimuthally integrated I(q) plots from the different tissue zones (SZ, TZ, DZ, CP and TB), corrected for diffuse background. I(q) plots are laterally averaged across sample width at specific vertical depths from cartilage surface; x-axis wavevector q is in nm-1 (B) Plots from (A) normalized to maximum peak intensity, to show the peak shape variations more clearly. (TIF) [file pone.0273832.s004.tif]

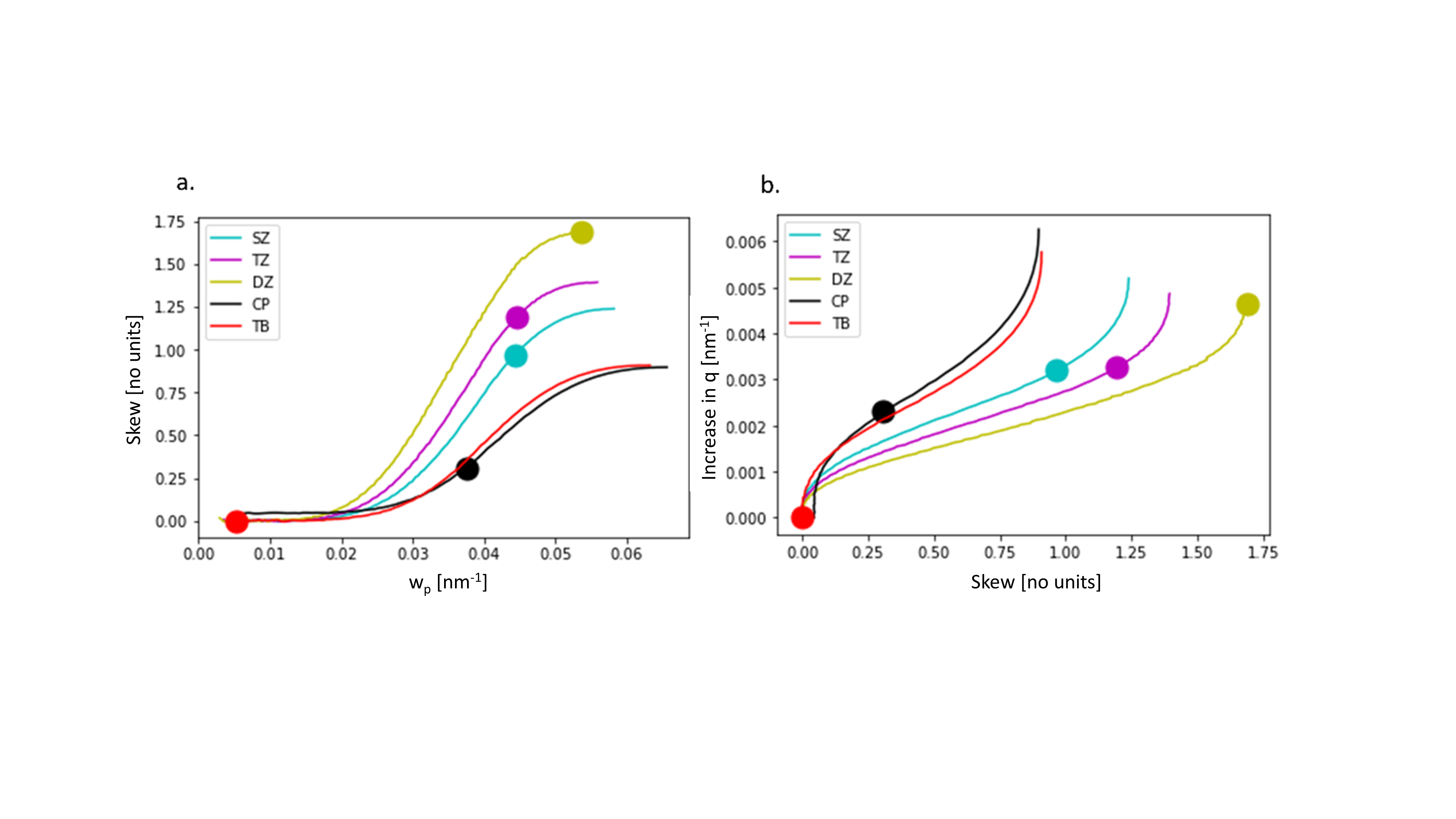

Supplement: S5 Fig — (A) Solid lines denote the simulated increase in skew as the equatorial width wp increases; as wa is different for each tissue zone, the curves are separate; wp is in units of nm-1. Filled circles indicate the experimentally determined skew (from S4 Fig) for each zone. (B) The predicted increase in peak position q0 with skew (in nm-1), as demonstrated in Fig 9 (main text); solid circles denote the expected increase for different tissue zones, using skew values obtained in (A). (TIF) [file pone.0273832.s005.tif]

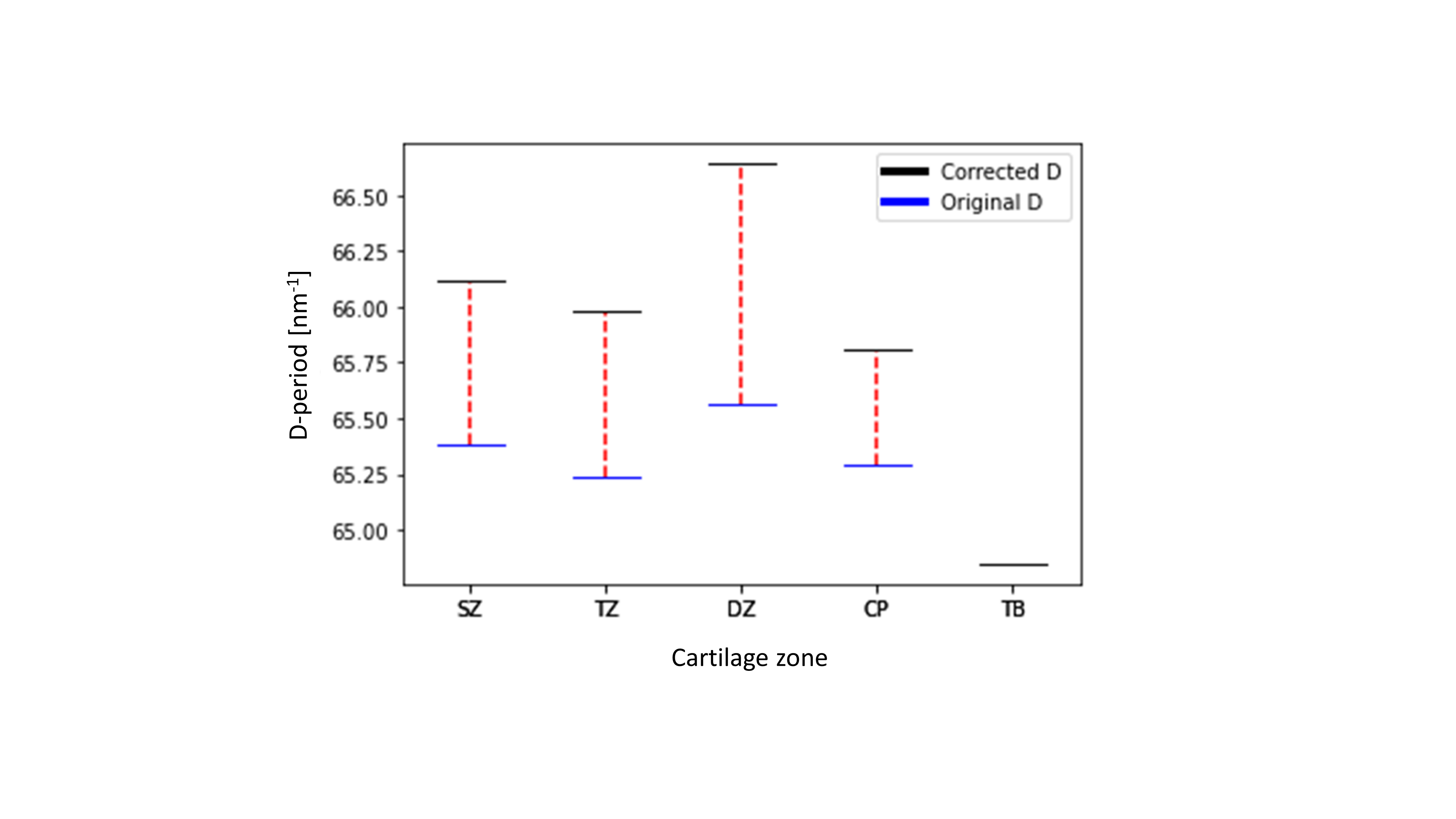

Supplement: S6 Fig — Blue bars: D-period (in nm) for the different I(q) curves in S4 Fig, calculated using the first moment of area method used in the main text. Black bars: True D-period, obtained by correcting for the artificial increase in D-period due to the skew, demonstrated in S5B Fig. (TIF) [file pone.0273832.s006.tif]
