# Supplementary material for: Collagen pre-strain discontinuity at the bone—Cartilage interface
Source: PLoS One. 2022 Sep 15;17(9):e0273832. doi: 10.1371/journal.pone.0273832 (PMC9477506; doi:10.1371/journal.pone.0273832)
Supplement: S1 Table — ANOVA tests followed by TUKEY HSD tests were carried out on the data. P values indicate statistical significance, where *** is (p<0.001), ** is (p<0.01), * is (p<0.05) and ns is non-significant difference between regions. (PDF) [file pone.0273832.s007.pdf]

Full-length scan							
Parameter	Anova	Tukey HSD					
	P value	Region	Diff	Lower	Upper	P adj	Signif
D-period	<2e-16	DZ-CP	-0.1088	-0.1743	-0.0434	0.0001	***
		TB-CP	-0.9740	-1.0306	-0.9174	0.0000	***
		TZ-CP	-0.3628	-0.4471	-0.2786	0.0000	***
		TB-DZ	-0.8651	-0.9034	-0.8268	0.0000	***
		TZ-DZ	-0.2539	-0.3272	-0.1807	0.0000	***
		TZ-TB	-0.6111	0.5457	0.6766	0.0000	***
ρ	<2e-16	DZ-CP	0.74466	-0.8142	2.3074	0.6080	ns
		TB-CP	-1.9789	-3.3292	-0.6284	0.0009	***
		TZ-CP	-3.3334	-5.3429	-1.3240	0.0001	***
		TB-DZ	-2.7255	-3.6387	-1.8122	0.0000	***
		TZ-DZ	-4.0800	-5.8259	-2.3341	0.0000	***
		TZ-TB	-1.3545	-2.9151	0.2060	0.1151	ns
Orientation	0.127	DZ-CP	-3.1012	-14.7246	8.5220	0.9025	ns
		TB-CP	-5.4598	-15.5161	4.5965	0.5021	ns
		TZ-CP	3.1326	-11.8311	18.0964	0.9497	ns
		TB-DZ	-2.3585	-9.1589	4.4418	0.8092	ns
		TZ-DZ	6.2339	-6.7672	19.2350	0.6061	ns
		TZ-TB	8.5924	-3.0289	20.2138	0.2279	ns

S1 Table. Results from statistical testing of each nanoscale parameter for differences in regions across the BCU for full-length scan. ANOVA tests followed by TUKEY HSD tests were carried out on the data. P values indicate statistical significance, where *** is (p<0.001), ** is (p<0.01), * is (p<0.05) and ns is non-significant difference between regions.
