# Supplementary material for: Collagen pre-strain discontinuity at the bone—Cartilage interface
Source: PLoS One. 2022 Sep 15;17(9):e0273832. doi: 10.1371/journal.pone.0273832 (PMC9477506; doi:10.1371/journal.pone.0273832)
Supplement: S2 Table — Data from the 6 regular scan samples. 1-way ANOVA test (with tissue zone as the factor) was carried out on the data, with each scan point labelled as belonging to a specific tissue zone. P values indicate statistical significance, where *** is (p<0.001), ** is (p<0.01), * is (p<0.05) and ns is non-significant difference between regions. (PDF) [file pone.0273832.s008.pdf]

| Regular scan | | | |
|---|---|---|---|
| **Parameter** | **Sample** | **Anova** | **Signif** |
| | | **P value** | |
| D-period | 1 | <2e-16 | *** |
| | 2 | <2e-16 | *** |
| | 3 | <2e-16 | *** |
| | 4 | <2e-16 | *** |
| | 5 | <2e-16 | *** |
| | 6 | <2e-16 | *** |
| ρ | 1 | 4.88E-13 | *** |
| | 2 | 4.99E-07 | *** |
| | 3 | 5.05E-16 | *** |
| | 4 | 0.167 | ns |
| | 5 | 0.00306 | ** |
| | 6 | 0.000191 | *** |
| Orientation | 1 | <2e-16 | *** |
| | 2 | 0.121 | ns |
| | 3 | <2e-16 | *** |
| | 4 | <2e-16 | *** |
| | 5 | <2e-16 | *** |
| | 6 | 1.45E-15 | *** |

**S2 Table. Results from statistical testing of each nanoscale parameter for differences across tissue regions in the BCU**. Data from the 6 regular scan samples. 1-way ANOVA test (with tissue zone as the factor) was carried out on the data, with each scan point labelled as belonging to a specific tissue zone. P values indicate statistical significance, where *** is ($p<0.001$), ** is ($p<0.01$), * is ($p<0.05$) and ns is non-significant difference between regions.
