# Supplementary material for: Collagen pre-strain discontinuity at the bone—Cartilage interface
Source: PLoS One. 2022 Sep 15;17(9):e0273832. doi: 10.1371/journal.pone.0273832 (PMC9477506; doi:10.1371/journal.pone.0273832)
Supplement: S1 Appendix — (PDF) [file pone.0273832.s009.pdf]

Collagen Pre-strain Discontinuity at the Bone-Cartilage Interface

Waqas Badar[1], Husna Ali[1], Olivia N Brooker[1], Elis Newham[1], Tim Snow[2], Nicholas J Terrill[2], Gianluca Tozzi[3], Peter Fratzl[4], Martin M Knight[1], and Himadri S Gupta[1]

[1] Institute of Bioengineering and School of Engineering and Material Science, Queen Mary University of London, London, E1 4NS, United Kingdom
[2] Harwell Science and Innovation Campus, Diamond Light Source, Harwell, Didcot, OX11 10 0DE, United Kingdom
[3] School of Engineering, London South Bank University, London SE1 0AA, United Kingdom
[4] Department of Biomaterials, Max-Planck-Institute of Colloids and Interfaces, Potsdam Wissenschaftspark, Golm, Germany

Fibre Diffraction Modelling:

Variation induced by first-moment D-period estimates:

The fibre-diffraction effect described in the Discussion leads to a rightward skew in I(q) (Fig 9). Here, we quantify the effect on measured D-period. S4a Fig plots radial intensity profiles of the 3rd order peak (after diffuse SAXS background subtraction) from the deep zone (DZ), calcified plate (CP) and trabecular bone (TB) regions from one of the regular 2D scans reported in the paper. S4b Fig shows the plots after normalisation to peak height, to show peak shape variations more clearly and correct for the much lower TB peak intensity.

The skew effect is much more pronounced in the cartilaginous (DZ and CP) regions, compared to the bone (TB) tissue. This is consistent with cartilage collagen fibrils being much smaller (larger w_p, Fig 9) than bone collage fibrils (e.g., Gottardi et al, *PLOS ONE* 11(10): e0163552; https://doi.org/10.1371/journal.pone.0163552), as w_p is inversely related to the fibril radius.

As a result of the skew, it is clear the first-moment method underestimates the D-period. A complete treatment would need a fitting of the SAXS scattering in both radial and azimuthal directions concurrently via a 3D model. However, to show that the main message of the paper (D-period values in articular cartilage and calcified plate exceed that of trabecular bone) is retained, we use the model sketched in Fig 9 to quantify the effect as follows:

- The axial width w_a is estimated for the three I(q) curves shown in S4 Fig, by calculating the HWHM for each peak on the left-hand side of the maximum, which is unaffected by the skew (as seen in Fig 9).

- For these three values of w_a, three separate curves are rendered in S5a Fig, showing how skew increases with w_p in each case. The three circles on the plots show the experimentally determined values of skew for the I(q) plots (DZ, CP and TB) in S4 Fig.
- S5b Fig plots, for these three cases, how the first-moment calculation of D-period varies as the skew increases. The three circles (one for each plot) indicate, likewise, the correction to D-period (note that decrease in D-period is plotted on the abscissa).

Using the information in S5b Fig, S6 Fig plots the uncorrected and corrected D-period values. Cartilaginous tissue D-period values increase consistently (SZ, TZ, DZ and CP), with the largest increase in the DZ, while trabecular bone values are nearly unchanged (as expected, as its skew is small). This showing that the difference between the unmineralized cartilage and calcified plate versus the trabecular bone is retained. Since we do not discriminate, in the calcified plate, between the calcified cartilage and the subchondral bone in the CP, it is possible the subchondral bone D-period values are lowering the CP average seen in S6 Fig.
