# Supplementary material for: Collagen pre-strain discontinuity at the bone—Cartilage interface
Source: PLoS One. 2022 Sep 15;17(9):e0273832. doi: 10.1371/journal.pone.0273832 (PMC9477506; doi:10.1371/journal.pone.0273832)
Supplement: Attachment — Submitted filename: Author_response_to_reviewers_comments_FINAL.docx [file pone.0273832.s010.docx]

**Author response to Reviewer comments “***Collagen Pre-strain Discontinuity at the Bone – Cartilage Interface***”:**

We thank both Reviewers for their supportive comments on the methodology and the manuscript, as well as their insightful and constructive comments and suggestions.

Below we list the comments in red and our response in blue. New text additions to the MS are indicated in yellow highlighted blue font in the current document. We have listed the comments as *R[Reviewer number]-[Comment number]*, i.e. R2-2 denotes the second comment of Reviewer 2.

We note that we have edited the order of words in the revised title (“Cartilage – Bone” to “Bone – Cartilage” to be consistent with usage in the main text.

Editor comments are prefixed by E, e.g. E1, E2, …

R1-1): The acronym AC is used early in the manuscript but, if I'm not mistaken, is defined only for the first time on line 293.

AR1-1): Acronym AC is now defined in line 54, where it is used for the first time.

*“On one side, there is a layer of articular cartilage (AC) with depth-dependent gradients in fibre orientation (first described by the Benninghof architecture* [1]*), type II collagen/proteoglycan content ratios, and hydration* [2]*, and (more recently identified) nanoscale collagen fibrillar pre-strain and intramolecular disorder* [3]*”.*

R2-1): Please check line 147 " ... using a high high-speed saw. "

AR2-1): We have removed the extra word **high.**

*“The metacarpal bone was removed, and two metacarpal condyles were isolated from each joint using a high-speed saw”.*

R2-2): Please improve the quality of Figures. The text in Figure 1 is difficult to recognize.

AR2-2): We have edited the quality of the figures, especially Figure 1 and enlarging the text within, and believe it is now improved.

R2-3): Please explain how the sample preparation method and the state of the sample during X-ray measuring might affect the experimental results? How to avoid these problems in this work?

AR2-3):

**Sample Preparation**:

If the samples are not kept hydrated during the extraction of BCU cores, the heating affect (due to friction) can damage the tissue and alter the mechanics. In order to minimize this effect of heating and mecahnical damage, the drilling system was used with constant PBS irrigation, with the drilling advancing slowly into the tissue under manual control.

**State of sample during X-ray measurement**:

During the SAXS scanning, 1) maintaining the sample in a **physiologically hydrated condition** and 2) minimizing possible **structural damage due to X-ray exposure** are the main factors to control for. These factors will potentially affect both the collagen structure at nanoscale in the BCU and alter the mechanics of the tissue as well. Both these effects lead to alterations in the structure which will manifest themselves as the alteration in the SAXS peak intensities and shapes.

1) Hydrated collagen shows higher odd-order intensities versus even-order intensities (2^nd^ order peak weaker than 3^rd^ order etc. [4]), while dehydrated collagen has the opposite behaviour. In our setup, all samples were immersed in saline in the fluid chamber during the measurements so remain fully physiologically hydrated, and this is confirmed by inspection of the SAXS peaks.
2) Radiation-induced structural damage can cause breakdown of the staggered arrangement inside collagen fibrils, leading to a reduction of the collagen peak intensity. In our experiment, each tissue location is exposed only once to the X-ray beam for 1.0s/point as part of a continuous scanning/rastering over the sample. To ensure that the single exposure did not result in structural damage, we carried out a radiation-damage test where a series of 1.0 s SAXS patterns were acquired continuously at a single tissue location in the deep zone of cartilage.

We observed reduction of peak intensity from the 4^th^ 1s SAXS pattern onward (4.0 s exposure), following which the SAXS peak intensity decreased to zero by the 20^th^ SAXS pattern (20s exposure), indicating structural breakdown by 20s. Thus, we expect that the single-exposure SAXS scan (1.0s/tissue location) is too short to cause radiation-induced structural damage.

Changes in text: The following text has been added at L593-L602, Discussion

“*… work. Thirdly, in terms of potential artefacts induced by sample preparation and experimentation, frictional heating during the coring process could damage the tissue and change the structure, and to prevent this, we use a slow coring under constant saline irrigation and cooling of the sample. To prevent any dehydration of the sample during the scanning (which will change the structure), the sample is kept fully immersed in physiological saline. Further, to minimize ultrastructural damage to the fibril structure due to X-ray radiation, in our protocol each tissue location on the sample is exposed only once to the beam (1.0s/point). Radiation-damage tests by repeated 1.0s SAXS exposures of the same cartilage tissue point show reductions of meridional peak intensity (characteristic of radiation damage) only after 4.0s of exposure, indicating that the single exposure scans are too short to cause damage. Fourthly, we …*”

E1): Please ensure that your manuscript meets PLOS ONE's style requirements, including those for file naming. The PLOS ONE style templates can be found at 
https://journals.plos.org/plosone/s/file?id=wjVg/PLOSOne_formatting_sample_main_body.pdf and 
https://journals.plos.org/plosone/s/file?id=ba62/PLOSOne_formatting_sample_title_authors_affiliations.pdf.

Please provide in the Methods section the name and location of the slaughterhouse from which samples were obtained.

AE1-):

The format (including Figure references, file naming, supplementary information) has been made consistent with PLOS ONE’s style requirements. The text in Supplementary Information is now reported as an appendix. The Figures and Tables in Supplementary Information are uploaded separately.

The name and location of the slaughterhouse from where the samples were obtained is added in the MS (lines 140-142)

*“BCU explants were extracted from a metacarpophalangeal (MCP) joint (****Figure 1C)****, of freshly slaughtered adult bovine steers (16-24 months of age), delivered by the local abattoir (C Humphreys & Sons- Blixes Farm Shop, Chelmsford, UK)”.*

E2): We note that you have stated that you will provide repository information for your data at acceptance. Should your manuscript be accepted for publication, we will hold it until you provide the relevant accession numbers or DOIs necessary to access your data. If you wish to make changes to your Data Availability statement, please describe these changes in your cover letter and we will update your Data Availability statement to reflect the information you provide.

AE2-): We have uploaded the data to the QMRO repository (<https://qmro.qmul.ac.uk/>). The DOI for the data is at: <https://doi.org/10.17636/10179773>

E3): Please amend either the title on the online submission form (via Edit Submission) or the title in the manuscript so that they are identical.

AE3-): Done; please note we have changed the order of “cartilage” and “bone” in the title to be consistent with the general notation “bone-cartilage interface (BCI)” in the manuscript.

E4): Thank you for stating the following in the Acknowledgments Section of your manuscript: 
[HSG (BB/R003610/1, EP/V011235/1) and NJT (BB/R003610/1, EP/V011235/1) thank the BBSRC and EPSRC for research grant funding. HSG thanks MRC (MR/R025673/1) for funding. We thank Diamond Light Source (DLS) for the generous award of synchrotron beamtime (SM25602-2).]

We note that you have provided funding information that is not currently declared in your Funding Statement. However, funding information should not appear in the Acknowledgments section or other areas of your manuscript. We will only publish funding information present in the Funding Statement section of the online submission form. 
Please remove any funding-related text from the manuscript and let us know how you would like to update your Funding Statement. Currently, your Funding Statement reads as follows: 
 [HSG: BB/R003610/1, EP/V011235/1, MR/R025673/1, SM25602-2
NJT: BB/R003610/1, EP/V011235/1, SM25602-2

BB/R003610/1: Biotechnology and Biological Sciences Research Council (https://www.ukri.org/councils/bbsrc/)
EP/V011235/1: Engineering and Physical Sciences Research Council (EPSRC) (https://www.ukri.org/councils/epsrc/)
MR/R025673/1: Medical Research Council 
(https://www.ukri.org/councils/epsrc/)
SM25602-2: Diamond Light Source (STFC) (www.diamond.ac.uk)
The funders had no role in study design, data collection and analysis, decision to publish, or preparation of the manuscript.]

Please include your amended statements within your cover letter; we will change the online submission form on your behalf.

AE4-):

We have removed funding information from the Acknowledgements as requested and added text thanking Dr. Smith and Dr. Shebanova (at Diamond Light Source) for technical assistance during the beamtime.

E5): Please review your reference list to ensure that it is complete and correct. If you have cited papers that have been retracted, please include the rationale for doing so in the manuscript text, or remove these references and replace them with relevant current references. Any changes to the reference list should be mentioned in the rebuttal letter that accompanies your revised manuscript. If you need to cite a retracted article, indicate the article’s retracted status in the References list and also include a citation and full reference for the retraction notice.

AE5-): References have been checked.

**References for Author response**:

1.	Benninghoff A. Form und Bau der Gelenkknorpel in ihren Beziehungen zur Funktion - Zweiter Teil: Der Aufbau des Gelenkknorpels in seinen Beziehungen zur Funktion. Zeitschrift für Zellforsch und Mikroskopische Anat. 1925;2: 783–862. doi:10.1007/BF00583443

2.	Lu XL, Mow VC. Biomechanics of articular cartilage and determination of material properties. Medicine and Science in Sports and Exercise. 2008. pp. 193–199. doi:10.1249/mss.0b013e31815cb1fc

3.	Inamdar SR, Barbieri E, Terrill NJ, Knight MM, Gupta HS. Proteoglycan degradation mimics static compression by altering the natural gradients in fibrillar organisation in cartilage. Acta Biomater. 2019;97: 437–450. doi:10.1016/j.actbio.2019.07.055

4.	Inamdar SR, Knight DP, Terrill NJ, Karunaratne A, Cacho-Nerin F, Knight MM, et al. The Secret Life of Collagen: Temporal Changes in Nanoscale Fibrillar Pre-Strain and Molecular Organization during Physiological Loading of Cartilage. ACS Nano. 2017;11: 9728–9737. doi:10.1021/acsnano.7b00563
